# Treatment of Urethral Pain Syndrome (UPS) in Sweden

**DOI:** 10.1371/journal.pone.0225404

**Published:** 2019-11-22

**Authors:** Lina Birgitta Ivarsson, Björn Erik Lindström, Matts Olovsson, Annika Kristina Lindström

**Affiliations:** 1 Department of Women´s and Children´s Health, Uppsala University, Uppsala, Sweden; 2 Center for Clinical Research Dalarna, Uppsala University, Uppsala, Sweden; 3 Clinical Research Center, Faculty of Medicine and Health, Örebro University, Örebro, Sweden; University Medical Center Utrecht, NETHERLANDS

## Abstract

**Background:**

Urethral Pain Syndrome (UPS) in women is a recurrent urethral pain without any proven infection or other obvious pathology. There are few studies on UPS, and evidence-based treatment is lacking. The primary aim was to study what treatments are used, and to compare the treatment tradition of UPS in Sweden in 2018, with what was used in 2006.

**Methods:**

A questionnaire on the treatment of women with UPS was sent to all public gynecology, urology, gynecologic oncology and venereology clinics, and one public general practice in each county in Sweden in 2018. Private practice clinics in gynecology responded to the survey in 2017. Comparisons were made with the same survey sent to gynecology and urology clinics in 2006.

**Findings:**

Of 137 invited clinics in 2018, 99 (72.3%) responded to the survey. Seventy-seven (77.8%) of them saw women with UPS and 79.2% (61/77) of these clinics treated the patients using 19 different treatment methods. Local corticosteroids and local estrogens were the methods most used. Treatments were similar in gynecology and urology clinics in 2006 and 2018, although strong corticosteroids had increased in use in the treatment regimens of 2018. More than half of the clinics used antibiotics.

**Interpretation:**

Since there is no evidence-based treatment of UPS, a wide spectrum of treatments is used, and different specialties use different treatment strategies. Despite the lack of proven infection, a large number of clinics also treated the syndrome with antibiotics. There is thus a need for well-designed randomized controlled clinical trials to find evidence-based treatments of UPS.

## Introduction

Urethral Pain Syndrome (UPS) in women is defined as the occurrence of recurrent episodic urethral pain, usually on voiding, with daytime frequency and nocturia, in the absence of proven infection or other obvious pathology [1]. The syndrome was formerly called urethral syndrome [2] or urethritis [3]. In 2002 the International Continence Society changed the terminology to “urethral pain syndrome” (UPS) as a part of the “genito-urinary pain syndromes” [1].

The UK Database of Uncertainties about the Effect of Treatments (DUET) has recognized Urethral Pain Syndrome (UPS) as a knowledge gap [4]. There are few randomized controlled trials and there is no evidence-based treatment of UPS [5, 6]. The syndrome is most frequent in women but is also seen in men and children [5]. UPS has a large impact on the patients’ quality of life; a study with clinical interviews showed higher levels of hostility, irritability, anxiety, dysphoria and depression among the UPS patients than in controls [7, 8]. The prevalence of UPS is unknown due to the lack of consensus on diagnosis and overlap with other diseases [5]. A study from England showed that around half of the patients visiting a general practitioner with frequency or dysuria, did not have any clinically significant bacteriuria [9]. Much controversy exists regarding the etiology of UPS. Previous studies indicate that the syndrome is not due to a single cause but to several complex mechanisms [5]. Low-grade infection, early interstitial cystitis (Painful Bladder Syndrome), urethral spasm, urethral stenosis, inflammation of the paraurethral glands (“female prostatitis”), estrogen deficiency in the urethral mucosa and psychogenic illness, have been investigated as possible causes [5, 10, 11]. Parson et al. reported a hypothesis concerning a common pathophysiological pathway behind the symptoms, regardless of etiology. Their hypothesis includes a dysfunction of the mucosal barrier that leads to inflammatory changes, a theory that is supported by several studies [5, 10, 12].

Many different treatment methods, including local corticosteroids, antibiotics, alpha-blockers, topical vaginal estrogen, female urethral dilation, local anesthetics, mucosal protecting agents, acupuncture, antidepressants, bladder training, and physical therapy, have been tested with various effects [5, 10, 13–16]. In previous studies, it is recommended that the therapeutic approach should be multimodal, and it is stated that “treatment at its best is by trial and error” [6, 10, 13]. This lack of evidence-based treatment of UPS results in many different treatments, depending on the individual physician’s preferred method and belief [15]. Initially, it is difficult to distinguish the UPS from urinary tract infection, which leads to unnecessary antibiotic treatment among UPS patients [9]. This common use of antibiotics in UPS, where there is no proven infection, needs to be addressed since it is most probably totally unnecessary and may also cause side effects and induce resistance to antibiotics [17]. The aim of this study was to analyze how clinics in Sweden treat women with UPS and to compare the results with the findings from a survey on treatment from 2006.

## Methods

In February 2018 a questionnaire regarding the treatment of women with UPS was sent to all hospital clinics in gynecology, urology, venereology, and gynecologic oncology, and one public general practice in each county of Sweden. The general practice was selected by contacting each county and asking for a large or medium-sized public general practice. The survey was sent to 137 clinics, of which 41 clinics were specialized in gynecology, 37 in urology, 11 in gynecological oncology, 27 in venereology, and 21 were primary care clinics. The questionnaire was first sent out as an electronic survey. The survey tool used was Textalk Websurvey (Memnon networks/Unifaun). A reminder was sent three weeks later to non-responders. In April the same questionnaire was sent by regular mail to the non-responding clinics with a reminder 3 weeks later. The questionnaire concerned the number of women with UPS that visited the clinic per month, whether the clinic treated these women or referred them somewhere else, whether the clinic had a treatment protocol regarding UPS diagnosing and treatment, and how the clinic treated women with UPS. The data were analyzed using Excel 2016, and data from 2018 were compared with data from a similar survey from 2006 that was sent to 30 hospital clinics (21 specialized in gynecology and 9 in urology). A survey of 57 gynecologists in publicly financed private practice was carried out in September 2017. An English version of the questionnaire is found in S1 Appendix.

There were no objections to the study protocol from the Regional Ethics Committee in Uppsala (reg. No 2017/561). The responders were informed of the study by the questionnaire and they gave their consent to take part in the study by completing and submitting the questionnaire.

## Results

Of the 137 clinics that were invited in 2018, 99 (72.3%) responded to the questionnaire. Thirty-five of the responding clinics were specialized in gynecology, 23 in urology, 20 in venereology, 6 in gynecological oncology, 14 were general practices, and 1 clinic did not state its identity. Seventy-seven (77.8%) of the responding 99 clinics had women with UPS at their practices, while 22 clinics (22.2%) did not see any UPS patients. Of the clinics that did see women with UPS, 79.2% (77/99) did treat these patients (Table 1).

**Table 1:** Clinics treating UPS.

Year	Medical speciality	Invited N	Answer n (%)	UPS patients visting n (%)	Treating UPS n (%)
2018	Gynecology	41	35 (85.4)	29 (82.9)	28 (96.6)
2018	Urology	37	23 (62.7)	18 (78.3)	15 (83.3)
2018	Venerology	27	20 (74.1)	14 (70.0)	8 (57.1)
2018	Oncology	11	6 (54.5)	4 (66.7)	2 (50.0)
2018	General Practitioner	21	14 (66.7)	12 (85.7)	8 (66.7)
2018	Not identified		1	0	0
	**Total 2018**	**137**	**99 (72.3)**	**77 (77.8)**	**61 (79.2**)
2017	Gynecology private*	**57**	**48 (84.2)**	**47 (98.0)**	**39 (85.1)**
2006	Gynecology	21	18 (85.7)	18 (100.0)	15 (83.3)
2006	Urology	9	9 (100.0)	9 (100.0)	8 (88.9)
	**Total 2006**	**30**	**27 (90.0)**	**27 (90.0)**	**23 (85.2)**

* Private practice, publicly financed

**Fig 1:**
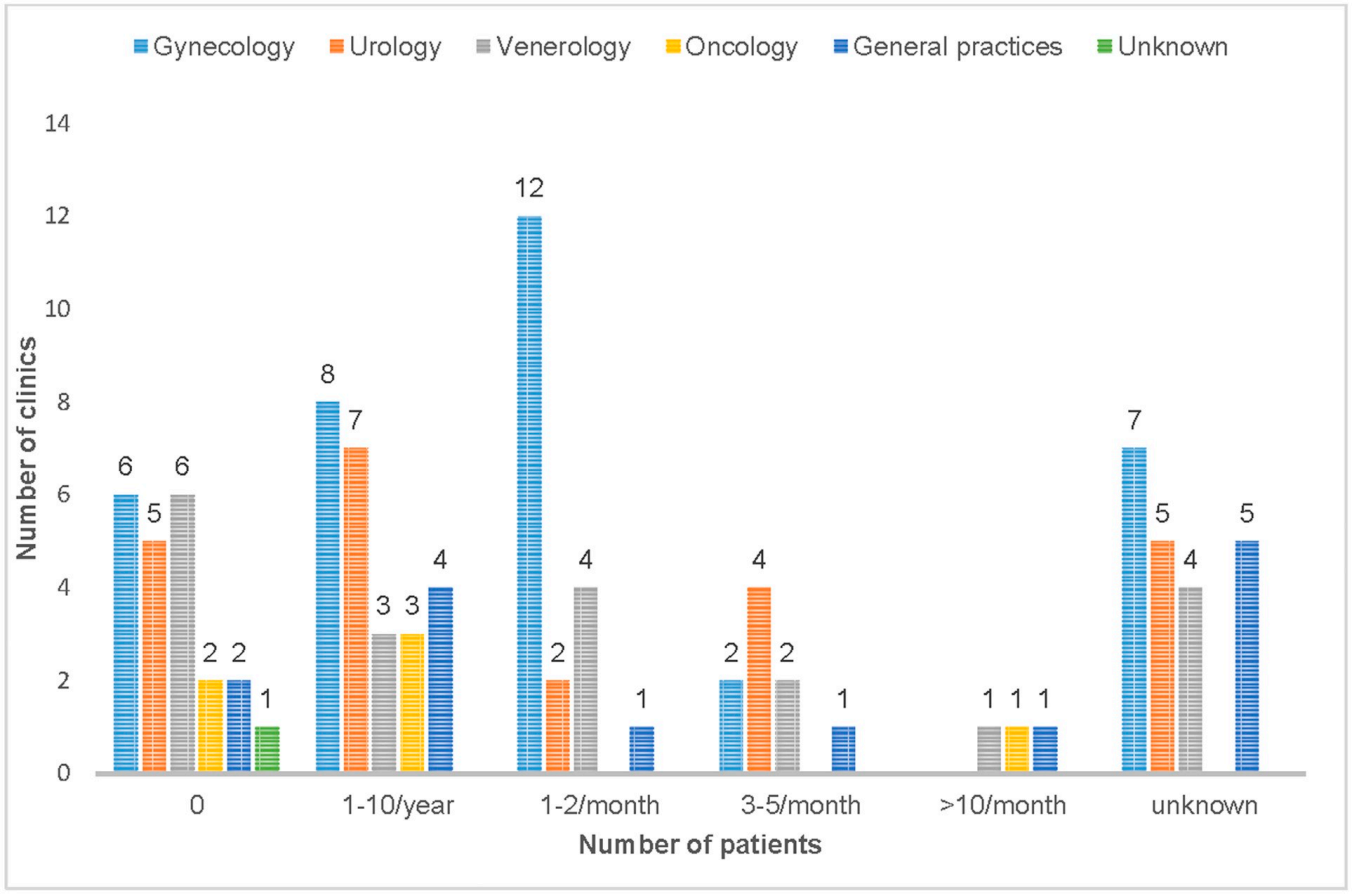
The number of UPS patients visiting different types of clinics (n = 99).

The oncology clinics treated patients with UPS secondary to radiotherapy of the pelvic area. The 16 clinics (21.1%, 16/77) that did not treat UPS, referred the patients to gynecology clinics, urology clinics, private clinics in gynecology or urology or other outpatient clinics. There was a treatment protocol for UPS at four clinics (4.0%); two specialized in gynecology, one in venereology and one was a general practitioner practice. The number of patients that visited the clinics is summarized in Fig 1.

The 61 clinics treating women with UPS used 19 different treatment methods (Table 2). The use of local corticosteroids was reported by the largest number of clinics and was the first line treatment in the majority of gynecology and urology clinics, alone or in combination with urethral dilation. Twelve clinics, 10 gynecology clinics, and 2 urology clinics, used corticosteroids with high or ultra-high potency. Oral antibiotics were reported as the first mode of treatment by the majority of venereology clinics, and local estrogen by oncology clinics and general practitioners.

**Table 2:** The number of clinics using different treatment methods to treat women with UPS in Sweden in 2018.

Treatment methods	All clinics	Gynecology	Urology	Venereology	Oncology	General Practice
	n (%)	n (%)	n (%)	n (%)	n (%)	n (%)
**Clinics treating UPS**	**61 (100)**	**28 (100)**	**15 (100)**	**8 (100)**	**2 (100)**	**8 (100)**
Local corticosteroids	42 (68.9)	26 (92.9)	13 (86.7)	1 (12.5)	2 (100.0)	0 (0.0)
Local estrogen	41 (67.2)	23 (82.1)	9 (60.0)	2 (25.0)	1 (50.0)	6 (75.0)
Urethral dilatation	30 (49.2)	18 (64.3)	12 (80.0)	0 (0.0)	0 (0.0)	0 (0.0)
Local anesthetics	22 (36.1)	14 (50.0)	5 (33.3)	1 (12.5)	1 (50.0)	1 (12.5)
Local antibiotics	16 (26.2)	10 (35.7)	4 (26.7)	0 (0.0)	1 (50.0)	1 (12.5)
Oral antibiotics	15 (24.6)	4 (14.3)	3 (20.0)	6 (75.0)	0 (0.0)	2 (25.0)
Silver chloride	10 (16.4)	8 (28.6)	2 (13.3)	0 (0.0)	0 (0.0)	0 (0.0)
Urethral massage	6 (9.8)	5 (17.9)	1 (6.7)	0 (0.0)	0 (0.0)	0 (0.0)
Oral estrogen	6 (9.8)	3 (10.7)	1 (6.7)	0 (0.0)	1 (50.0)	1 (12.5)
Physiotherapy	2 (3.3)	1 (3.6)	1 (6.7)	0 (0.0)	0 (0.0)	0 (0.0)
Antidepressants	2 (3.3)	1 (3.6)	0 (0.0)	0 (0.0)	1 (50.0)	0 (0.0)
Botox	1 (1.6)	1 (3.6)	0 (0.0)	0 (0.0)	0 (0.0)	0 (0.0)
Oral COX-2 inhibitors	1 (1.6)	1 (3.6)	0 (0.0)	0 (0.0)	0 (0.0)	0 (0.0)
Urotheraphy	1 (1.6)	0 (0.0)	1 (6.7)	0 (0.0)	0 (0.0)	0 (0.0)
Mucosal protectants	1 (1.6)	0 (0.0)	1 (6.7)	0 (0.0)	0 (0.0)	0 (0.0)
Silver nitrate	1 (1.6)	0 (0.0)	1 (6.7)	0 (0.0)	0 (0.0)	0 (0.0)
Alpha blockers	1 (1.6)	0 (0.0)	1 (6.7)	0 (0.0)	0 (0.0)	0 (0.0)
Spasmolytics	1 (1.6)	0 (0.0)	0 (0.0)	0 (0.0)	1 (50.0)	0 (0.0)
Other	1 (1.6)	1 (3.6)	0 (0.0)	0 (0.0)	0 (0.0)	0 (0.0)

In September 2017 a paper survey of the treatment of UPS was distributed to 57 gynecologists working in publicly financed private practice, and 49 answered the questionnaire (86.0%). Of the 48 gynecologists who had patients with UPS, seven did not treat the syndrome but referred the patient to a colleague (six to a urologist and one to a gynecologist). However, these gynecologists often initiated local estrogen treatment for the patient before referral. Fourteen gynecologists administered all the treatments themselves, while 27 sometimes referred to a colleague for further treatment (urologist 22, gynecologist 5 and urotherapist 2). Half of the gynecologists (44/48) found UPS to be a common problem. Fourteen different treatments, alone or in different combinations, were used (Table 3).

**Table 3:** Private practitioners in gynecology using different treatment methods to treat women with UPS in Sweden 2017.

Treatment methods	Gynecology
	n (%)
**All Clinics**	**41 (100.0)**
Local estrogen	39 (95.1)
Local corticosteroids	23 (56.1)
Urethral dilatation	17 (41.5)
Oral antibiotics	15 (36.6)
Local antibiotics	15 (36.6)
Psychological support	15 (36.6)
Oral estrogen	12 (29.3)
Antidepressants	12 (29.3)
Urethral massage	11 (26.9)
Silver chloride	8 (19.5)
Acupuncture	3 (7.3)
Oral corticosteroids	2 (4.9)
Policresulen	1 (2.4)
Metenaminhippurat	1 (2.4)

The questionnaire that was sent to 21 gynecology and 9 urology clinics in 2006 had a 90% response rate. Of the responding 27 clinics, 18 (66.7%) were gynecology clinics and 9 (33.3%) urology clinics. Twenty-three clinics (85.2%), 15 gynecology clinics and 8 urology clinics, treated the UPS. The clinics used, at this time, 17 different treatment methods (Table 4). Local corticosteroids and local estrogen were the first line treatments at most clinics.

**Table 4:** The number of clinics using different treatment methods for UPS in Sweden 2006.

Treatment methods	All clinics	Gynecology	Urology
	n (%)	n (%)	n (%)
**Clinics treating UPS**	**23 (100)**	**15 (100)**	**8 (100)**
Local corticosteroids	18 (78.3)	10 (66.7)	7 (87.5)
Local estrogen	17 (73.9)	9 (60.0)	7 (87.5)
Urethral dilatation	13 (56.5)	7 (46.7)	6 (75.0)
Local antibiotics	10 (43.5)	7 (46.7)	3 (37.5)
Local anesthetics	9 (33.3)	6 (40.0)	3 (37.5)
Oral antibiotics	6 (26.1)	4 (26.7)	2 (25.0)
Urethral massage	6 (26.1)	5 (33.3)	1 (12.5)
Silver chloride	4 (17.4)	2 (13.3)	2 (25.0)
Oral COX-2 inhibitors	4 (17.4)	2 (13.3)	2 (25.0)
Oral estrogen	4 (17.4)	3 (20.0)	1 (12.5)
Acupuncture	2 (8.7)	0 (0.0)	2 (25.0)
Silver nitrate	2 (8.7)	1 (6.7)	1 (12.5)
Electrical stimulation	2 (8.7)	0 (0.0)	2 (25.0)
Policresulen	1 (4.3)	0 (0.0)	1 (12.5)
Metenaminhippurat	1 (4.3)	1 (6.7)	0 (0.0)
Chondroitin	1 (4.3)	1 (6.7)	0 (0.0)
Psychological support	1 (4.3)	1 (6.7)	0 (0.0)

Some commentaries from the clinics are:”exclude sexual abuse”, “we have tried many treatments over the years but have no good therapies”, “advice on clothing and hygiene products”, “heat helps some patients”, “good care of the patient is important”,”physiotherapy if symptoms of pelvic pain” and “patients are sometimes also seeing a medical councilor or midwife as a pain patient”.

## Discussion

UPS remains a difficult dilemma for the practicing physician as there is a lack of evidence-based treatment. The major findings in this study were that there is a large variety of treatments of UPS in Sweden, and that more or less the same methods were used in 2018 as in 2006. The 2018 questionnaire showed that UPS patients were seen and treated at all medical specialty departments (urology, gynecology, gynecologic oncology, venereology, and primary care) included in the survey. In the 2018 survey, at the 61 clinics where UPS was treated, there were 19 different treatments reported, as compared to 17 in the 2006 survey, and 12 by private gynecologists in 2017. The various medical specialists preferred different methods, urologists and gynecologists primarily used local corticosteroids, in some clinics in combination with urethral dilation, whereas general practitioners, oncologists, and venereologists mainly used estrogen and antibiotics. Only four clinics had a treatment protocol regarding UPS.

### Corticosteroids

Local corticosteroids were the most common treatment by gynecologists, urologists, and oncologists in 2018, while they do not seem to be used by general practitioners. Oral corticosteroids were used by a few urologists and gynecologists in 2006 and by a few private practitioners. Inflammatory changes in the urethral mucosa have been detected in women with UPS, and inflammation is assumed to be a part of the pathophysiology behind the syndrome [5, 12, 14]. Instillations of local corticosteroids in the urethra could reduce the patient’s symptoms due to its anti-inflammatory effect. In 2006 the clinics used mainly topical corticosteroids of low potency and in 2018, 10 gynecology clinics and 2 urology clinics used topical corticosteroids of ultra-high potency in their treatment of women with UPS. Thus far, there are few studies where local treatment with corticosteroids has been investigated. In 1971, Nilsson published a study showing better results when combining urethral dilation with local low potent corticosteroids, compared with urethral dilation alone [18]. Lindström and co-workers reported positive effects of instillations of ultra-high potency glucocorticoid, clobetasol-propionate, combined with the local anesthetic lidocaine, in women with UPS [14].

### Estrogens

According to the 2018 questionnaire, the second most common treatment method was vaginal estrogen, which was mainly used by gynecologists in private practice. To date, there are several studies on estrogen treatment of symptoms from the lower urinary tract. At a sufficient dose, vaginal estrogen can affect the urethral epithelium and strengthen the mucosal barrier, thereby reducing the irritative symptoms from urinary frequency and incontinence [19, 20]. Youngblood and co-workers stated as early as 1957 that topical vaginal estrogen improves urethritis in postmenopausal women [3]. However, in a study including patients of all ages with urethral syndrome, no benefit was found from treating UPS patients with estrogen combined with wide-spectrum antibiotics, compared to wide-spectrum antibiotic treatment alone [21]. Vaginal estrogen should probably be first-line therapy for postmenopausal women with UPS. There are no studies on oral estrogens and UPS. In two Cochrane reports on estrogens and lower urinary tract symptoms (LUTS), such as urinary incontinence and recurrent urinary infection, an efficient dose of local estrogen treatment has a significant effect, but oral estrogens do not [22, 23].

### Urethral dilatation

Urethral dilation constitutes a controversial treatment. Despite this, our survey showed that urethral dilation was commonly used. Half of the clinics declared actually using urethral dilation in the surveys of 2006 and 2018, as did more than one-third of the private gynecologists. The procedure was exclusively performed by gynecologists and urologists and often in combination with local corticosteroids and massage [18, 24]. Urethral stenosis has been proposed as a possible etiology for UPS [24]. It has been suggested that urethral dilatation causes a kind of massage of the mucous membranes that stretches the submucosal tissue and subsequently remedies strictures. It is also thought to relieve meatal stenosis and to promote laminar flow. Another benefit from urethral dilation could be drainage of small abscesses in the paraurethral glands [21]. Bergman et al. found symptom relief in 75% of the patients after repeated urethral dilations, and suggested that serial urethral dilations are more effective than tetracycline and placebo in treating UPS [25]. Other studies state, on the other hand, that urethral dilation as a treatment of UPS should be limited. Yoon et al. reported various complications with urethral dilation and suggested that it only improves symptoms in patients with urethral obstruction, which is present in a few UPS patients [15]. In 2013, Bazi and co-workers supported Yoon’s statement and suggested that there is no justification for urethral dilation for the treatment of UPS, and that this procedure may do more harm than good. Urethral dilation is an invasive surgical procedure that causes irritative symptoms and is associated with various complications, including infections, incontinence and stricture development [16].

### Local anesthetics

According to the questionnaires, use of local anesthetics was also a common treatment for UPS. In a pilot study on the treatment of UPS, lidocaine was added to the local treatment of clobetasol-propionate [14]. We found no other published data on the use of local anesthetics for the treatment of patients with UPS, but there is a paper published by Parsons and co-workers where they could show symptom relief with mixtures of heparin and lidocaine for the treatment of interstitial cystitis [26, 27].

### Antibiotics

Despite the fact that a criterion for UPS is the absence of proven infection, antibiotics are commonly used. In this study, local antibiotics were used by about one-third of the responders, and by all medical specialties to treat UPS. Any use of antibiotics, oral or local, was the case in about half of the clinics. A low-grade infection that leads to mechanical and neurological changes is suggested as a possible etiology of UPS. Burkhard and co-workers claim that cystoscopy of UPS patients often reveals signs of chronic infection, and Parziani and co-workers reported higher rates of *Ureaplasma urealyticum* and *Chlamydia trachomatis* in UPS patients compared with controls [21, 28]. Kyndel and co-workers found, however, no evidence that *Ureaplasma* or *Mycoplasma* colonization was more prevalent in women with UPS than in asymptomatic women [29]. To date, there are several studies with various results where antibiotic treatments have been investigated for the treatment of UPS patients. Kaur and co-workers state that antibiotics should be used as first-line therapy for patients with UPS [13]. Burkhard and co-workers suggest that doxycycline treatment is justified, since 70% of their study population did benefit from treatment with doxycycline [28]. Bergman and co-workers reported, however, no greater cure rate with tetracycline therapy than placebo [25]. Tetracyclines have an anti-inflammatory effect alongside the antiseptic effect, and that might explain the results [30]. New data support the fact that the treatment of painful LUTS associated with pyuria with very long-term antimicrobial courses, despite negative urine culture, is effective [31]. This type of long-term treatment of LUTS is under debate [32].

### Silver chloride

Silver chloride instillations were used to treat UPS by 15–20% of respondents in this study. The medicinal use of silver is of ancient origin and solutions of silver chloride have been used for bladder instillations [33]. We found no studies on the use of silver chloride for the treatment of UPS.

### Urethral massage

Urethral massage was used by about 20% of the gynecology and urology clinics, two of the clinics used it alone or combined with dilatation or a drug. The rationale and technique of urethral massage are described by Scotti [24]. There are no studies on the effect of massage of the urethra in the treatment of UPS.

### Psychological support

Patients with UPS have been considered to have a psychological component, but possibly they are merely stressed by the continuing symptoms and inadequate diagnosis and treatment [5]. A study from 1989 showed more symptoms of psychological distress in the group of patients with urethral syndrome than in the control group [7]. Another study by the same authors highlighted the appearance of the urethral syndrome in highly stressful situations and an increase in the patient’s psychological symptoms [8]. Other studies demonstrated no significant differences in depression or anxiety among those with urethral syndrome compared to a control group [34, 35]. Psychological support was given by more than one-third of gynecologists in private practice and reported by one gynecology clinic in 2006.

### Physiotherapy, urotherapy

The UPS is associated with other genitourinary pain syndromes such as pelvic pain syndrome, and a pilot study suggests that physiotherapy, i.e. palpation of trigger points and massage of the pelvic floor musculature, could relieve symptoms of UPS when pelvic floor tension is present [36]. Urotherapy and bladder training could improve patients voiding habits [15]. Physiotherapy and urotherapy were used by 3 clinics in 2018.

### Antidepressants

Tricyclics antidepressants have been shown to have an analgesic effect and can give pain-relief in various chronic pain syndromes [37]. A large randomized, placebo-controlled study on the treatment of interstitial cystitis showed, nonetheless, no difference between the tricyclic amitriptyline and placebo [38]. UPS is stressful in itself and also has a tendency to occur during stressful periods in life [5, 13]. Antidepressants were mainly used by private gynecologists who often have better opportunities to maintain continuity in patient follow-up.

### Silver nitrate

In the pre-antibiotic era, gonorrhea was treated with injection therapy or topical treatment with a solution of silver nitrate on the mucosa of the urethra [39]. In a publication from Sweden 1975, Ingelman-Sundberg describes the treatment of non-infectious urethritis with careful dilatation of the urethra and massage, in combination with local treatment of the mucosa using 1% solution of silver nitrate [40]. Very few gynecologists and urologists use silver nitrate therapy today.

### Acupuncture

In a small study, electric acupuncture had a better therapeutic effect on women with urethral syndrome than the oryzanol and diazepam given to a control group [41]. Another study in Traditional Chinese Medicine showed the effect of acupuncture and moxibustion therapy [42]. Acupuncture was given by two urology clinics in 2006 and by three gynecologists in private practice.

### Botulinum toxin

The neuromuscular blocker botulinum toxin has a wide variety of medical applications and off-label use in urology, including detrusor external sphincter dyssynergia and pelvic pain syndromes [43, 44]. It was used by one gynecology clinic in 2018.

### Alpha-blockers

A case report showed symptom relief from treatment with alpha-adrenergic blocking agents in one woman [45]. We found no other relevant studies for this regime. One urology clinic used alpha-blockers in 2018.

Treatment with electric stimulation, policresulen, COX-3 inhibitors, spasmolytics, and mucosal protectants, were reported by single clinics. However, we found no relevant studies on the use of these treatments of UPS.

There is thus a large range of treatments used for women with UPS. There is however no scientific evidence for any of them when it comes to clinical effect. Women with UPS often have to try many different treatments before achieving any symptom relief, as is shown in this study where patients were often referred to other clinics when treatment had been unsuccessful. As psychosocial aspects are involved in the disease, it is important to treat the patient by giving a lot of time, attention and psychosocial support during treatment.

A strength of the current study is the high response rate to the questionnaires. The overall response rate to the survey in 2018 was 71.5%. Gynecology clinics have many patients with UPS and a response rate as high as 85.4%. Because of the high response rate and the fact that the questionnaire was sent to all public gynecology, urology, venereology and gynecologic oncology clinics in Sweden, we believe that our results can be generalized, at least to Swedish inpatient clinics.

A limitation of the study is that only one public general gynecological practice in each county was approached in our survey and that their response rate was only 61.9%. In this case the results are most probably not generalizable to all Swedish general practices. Women with LUTS tend to seek help at their local health centers, and thus general practices are often the clinics that first come in contact with UPS patients, and therefore receive a large number of women with UPS.

Examples of other treatments of the UPS that have not been used by these clinics are cryosurgery [46], surgery [47], Yag laser [48] or phytotherapeutic products [49].

## Conclusion

Women with UPS are treated with many different methods, and various medical specialties have different ways to approach the syndrome. In urology and gynecology clinics the treatments used were more or less the same in 2018 as in 2006. The many different treatment modalities reflect the lack of evidence-based treatment. “As instillations of corticosteroids in the urethra is a common treatment in Sweden we suggest a Phase II study on the safety of a strong corticosteroid in the urethra at first and then a randomized controlled trial as a Phase III b trial. If this treatment shows clinical effect it will be an evidence based treatment on label for the corticosteroid studied.

Well-designed randomized controlled clinical trials are warranted to put the treatment of UPS into the era of evidence-based medicine.

## Supporting information

S1 Appendix Survey treatment of UPS. (PDF)

S1 File Treatment of UPS. (XLSX)
