# Supplementary material for: Treatment of Urethral Pain Syndrome (UPS) in Sweden
Source: PLoS One. 2019 Nov 22;14(11):e0225404. doi: 10.1371/journal.pone.0225404 (PMC6874337; doi:10.1371/journal.pone.0225404)
Supplement: S1 Appendix — (PDF) [file pone.0225404.s001.pdf]

Survey on the treatment of Urethral Pain Syndrome

1. Name of clinic: …………………………………………………………………………………………..

2. Do you have a care program for the treatment of UPS at your clinic? ☐ Yes ☐ No

3. Do women with UPS visit your clinic? ☐ Yes ☐ No

4. How many women with UPS visit your clinic? ☐ 1-10/year ☐ 1-2/month ☐ 3-5/month ☐ 5-10/month ☐ >10/month ☐ Do not know

5. Do you treat women with UPS? ☐ Yes ☐ No

If the answer to question 5 is No →

6a. For what reason do you not treat women with UPS? ……………………………………………….

……..

6b. Do you refer these patients to other caregivers (state which): ………………………………………..

If the answer to question 5 is Yes →

7a. What treatment is carried out? **Please cross all your treatment alternatives.**

☐ A. Urethral dilatation – type and size of dilator/probe/catheter: …………………………………...

☐ B. Massage of the urethra

☐ C. Local anesthesia – type of local anesthesia: ………………………………………………...

☐ D. Corticosteroids local – type of corticosteroid: ……………………………………......................

☐ E. Corticosteroid oral – type of corticosteroid: ……………………………………...................

☐ F. Instillation of silver chloride

☐ G. Albothyl© treatment (earlier Nelex©) of the urethra

☐ H. Instillation of silver nitrate

☐ I. Antibiotics oral – type of antibiotic: ……………………………………...

☐ J. Antibiotics local – type of antibiotic: ……………………………………..

☐ K. Estrogen oral – type of estrogen: …………………………………………

☐ L. Estrogen local – type of estrogen: ……………………………………………

☐ M. Others:
…………………………………………………………………………………...

……………………………………………………………………………………………

……………………………………………………………………………………………

7b. First line of treatment (single or in combination) according to letters above: ..……………………

7c. Is there anything you wish to add concerning your treatment of UPS in women?

……………………………………………………………………………………………………..

……………………………………………………………………………………………………..

……………………………………………………………………………………………………..

……………………………………………………………………………………………………..

Thank you very much for your help!
